# From Verbal Account to Written Evidence: Do Written Statements Generated by Officers Accurately Represent What Witnesses Say?

**DOI:** 10.3389/fpsyg.2021.774322

**Published:** 2022-02-10

**Authors:** Rebecca Milne, Jordan Nunan, Lorraine Hope, Jemma Hodgkins, Colin Clarke

**Affiliations:** ^1^Institute of Criminal Justice Studies, University of Portsmouth, Portsmouth, United Kingdom; ^2^Department of Psychology, University of Portsmouth, Portsmouth, United Kingdom

**Keywords:** witness, investigative interviewing, evidence, consistency, statements

## Abstract

Most countries compile evidence from witnesses and victims manually, whereby the interviewer assimilates what the interviewee says during the course of an interview to produce an evidential statement. This exploratory research examined the quality of evidential statements generated in real world investigations. Transcribed witness/victim interviews (*N* = 15) were compared to the resultant written statements produced by the interviewing officer and signed as an accurate record by the interviewee. A coding protocol was devised to assess the consistency of information between what was said by the interviewee in the verbal interview and what was reported in the written statement. Statements contained numerous errors including omissions, distortions, and the inclusion of information not mentioned in the verbal interview. This exploratory work highlights an important area for future research focus.

## Introduction

Witnesses are central to most criminal cases; indeed, some have argued they provide the most critical evidence in court (Zander and Henderson, 1993). Consequently, considerable attention has been paid to developing techniques that elicit reliable, relevant, and detailed information from witnesses during interviews (Gabbert et al., 2016; Milne and Bull, 2016). Traditionally, witnesses provide their accounts at two separate points of the criminal justice process; first when interviewed during the investigation and later when giving evidence during criminal proceedings (Westera et al., 2011). The information provided initially as part of the investigation not only informs investigative decision making (e.g., what lines of inquiry to pursue and prioritize), it is also central to legal decision-making, for example, whether to proceed with the case (or not). The written statement, produced when the interviewer assimilates the information provided by the witness in the course of the interview, is also key in any resultant court-case, informing legal strategy and likely serving as a memory aid for the witness. Clearly, the written product of the witness interview should thus be an accurate representation of what the witness reports about the event in question. The criminal justice system relies on the accuracy of this statement to avoid ill-informed investigative and legal decisions. This exploratory research examined the quality of evidential statements taken in real world investigations and, specifically, assessed the extent to which the written statements produced were in fact consistent with the content of the associated verbal interviews.

The purpose of an investigation is to establish what, if any, criminal offending has taken place and the identity of those who may be culpable (Milne and Bull, 2006). To answer these primary investigative questions the police seek information from a number of sources, including witnesses. The most common way to formalize witness accounts across the world is for an officer to produce a written (hand-written or typed) statement reflecting the information obtained during an interview. Statement production is often conducted at the same time as interviewing the witness, however this is dependent on circumstance (e.g., dynamic nature of the event), crime type (e.g., seriousness of the offense), officer training, and individual preference, i.e., there is limited evidenced based practice guidance (though see Smith and Milne, 2018 for a United Kingdom example- WISCI- Witness Interview Strategies for Critical Incidents). After being endorsed and signed by the witness, the statement is then used as the basis for investigative and legal decision-making. Often, the interview or the process of transferring the verbal content of that interview into a written statement is not electronically recorded or otherwise documented.

To date, psychological research has concentrated on enhancing our understanding of how the interview process can affect a witness’s memory recall of events and the development of techniques to enhance the quality and quantity of information obtained in witness interviews (Vrij et al., 2014). These advances have influenced police practices in many jurisdictions (Milne et al., 2019) and there is now growing consensus with respect to witness interviewing best practice (see Meissner, 2021 and associated special issue). Interviewers are encouraged to start such interviews with a free recall, followed by open-ended prompts and questions, and finishing with appropriate non-leading closed questions if necessary (see e.g., Achieving Best Evidence Guidance, Home Office, 2011). Open questions such as “tell me what happened…” are generally considered the best type of question to use because they encourage a detailed and unrestricted answer. As questions become more specific or interviewer-driven, responses become less accurate (Oxburgh et al., 2010; Boon et al., 2020; Kontogianni et al., 2020). In practice however, the usual method of recording the witness-police interaction relies on the interviewer’s own memory of what the witness said and there is typically no actual record of the questions used by the interviewer to obtain the witness’s account.

Indeed, Barristers Heaton-Armstrong and Wolchover (1992) were one of the first to argue that written statements are mistakenly treated by the criminal justice system as a verbatim record of interview:

*“There is a certain coyness on the part of most officers, when asked how they “took” a statement, in admitting that the narrative was obtained by questioning. The fiction is perpetuated that for the most part statements are the product of straight dictation.” (p. 161).*

The production of a written statement involves the interviewer, both deliberately and inadvertently, filtering the information generated by the witness during the interview, and deciding what should and should not be included in the statement (Westera et al., 2011). The cognitive demands of this task make it susceptible to distortion at many stages and the resulting statement is an abridged and often inaccurate version of what was said within the interaction. Further, in the United Kingdom (and many other countries) there is no legal requirement to make a record of the utterances of the interviewer (e.g., questions used) within the resultant statement. Given the importance of witness statements within the criminal justice system, there has been very limited research examining the accuracy of this witness statement-taking process.

Kohnken et al. (1994) examined the statement-taking process in a mock-witness experimental paradigm and found statements written by the interviewer immediately after the interview contained only about two thirds of the information reported by the witness. Similarly, Hyman Gregory et al. (2011) examined notes made by 13 US police investigators during a single mock witness interview and compared them to their subsequent reports. This comparison revealed that 68% of the information reported by the witness was omitted with 40% of the omitted information being deemed crime-relevant. In a US sample of 20 real-life interviews with child witnesses/victims, Lamb et al. (2000) found the interviewer’s “verbatim” notes were missing 25% of the forensically relevant details reported by the witness. In the United Kingdom, McLean (1995) examined 16 formal witness-police interviews and found that none of the statements contained all the relevant information reported by witnesses. These types of omission errors may be due to the cognitive load inherent in the multitude of tasks that constitute the statement taking process, for example, actively listening to the interviewee, formulating which questions to ask, assimilating the information reported, and taking comprehensive notes (Fisher et al., 2014; Kleider-Offutt et al., 2015; Hanway et al., 2021). Indeed, the cognitive load associated with the conduct of interviews is well recognized by police interviewers (Hanway and Akehurst, 2018). One possible result of reduced cognitive resources is that interviewers may, unwittingly, prioritize information that fits with their existing expectations or schema for the reported event. When information provided by a witness is not consistent the interviewer can: (i) include the information in full; (ii) distort the information to make it more consistent, or (iii) omit the information altogether (McLean, 1995). Furthermore, and worryingly, it would appear that witnesses fail to detect such revisions or errors in their own statements (Sagana et al., 2017).

Using cases drawn from two forces in the United Kingdom, the current research examined the consistency between information provided in verbal interviews with the resultant evidential statement. Specifically, we sought to identify any inconsistencies emerging in this translative process and describe the nature of those inconsistencies using a comprehensive coding protocol.

## Materials and Methods

### Case Materials

As part of the national evaluation of PEACE in the United Kingdom (Clarke and Milne, 2001) police officers were asked to record their interviews with real-life witnesses/victims, including the statement taking segment of the interview. Six forces (of 43 force areas) in England and Wales agreed to participate in the research. In order to gain a representative sample across the country, forces were selected based on willingness to participate, geographical location, and size of force (for a full outline of the National evaluation, see Clarke and Milne, 2001). At the time, two forces also gathered the resultant hand-written statement and submitted them to the research team as part of the project materials, but these were not included as part of the original evaluation, which focussed on the quality of the interview process. For the current research, 15 cases where the recorded interview with a witness including the statement-taking segment and the resultant hand-written statement were available and were analyzed. The cases analyzed included ten thefts, three criminal damage cases, one assault, and one public order incident. The statement length varied in length from 1 to 6 pages (*M* = 2.7, *SD* = 1.4).

### Coding Protocol

Drawing on the existing literature on consistency across reporting in investigative settings (e.g., Fisher et al., 2009), a coding protocol was developed to determine the extent to which what the witness reported in the interview was consistent with what the officer recorded as their evidence, at the time, in the form of a hand-written statement.

The following categories were included in the coding protocol: (i) consistent details (mentioned by the interviewee and included in the hand-written statement); (ii) omissions (mentioned by the interviewee and omitted from the hand-written statement); (iii) distortions (mentioned by the interviewee and written down incorrectly by the interviewer); (iv) contradictions (written in the statement but directly contradicts what was said by the interviewee), and (v) intrusions (not mentioned by the interviewee at any point but included in the statement). Omissions, distortions, contradictions and intrusions all reflect error in the translation of a verbal account into a written statement. We also coded for the category “known information” which reflects factual information known to the interviewer (mainly demographic) but not necessarily mentioned in the interview (e.g., address of interviewee). Following common interview coding approaches (e.g., Milne and Bull, 2002; Gabbert et al., 2009), each category was also coded with respect to type of detail i.e., persons, actions, objects, surroundings, conversation, and temporal information.

The second author coded the data, which comprised 15 hand-written statements and their partnering interview transcripts for comparison. The procedure involved comparing each hand-written statement to the counterpart transcript of what the witness actually said within the interview. Firstly, the coder examined each detail in the transcript and ascertained whether it was included in the statement. If not, then it was coded as an omission (1 point per item of information). If it was included within the statement then it was determined if it was included accurately and coded as either, “known,” “consistent,” “distortion” or “contradictory” (1 point per item of information). Any information within the statement but not in the transcript, was coded as an inclusion (1 point per item of information). The final step was to code each piece of information with respect to detail type- person etc. (as outlined above). An independent coder was randomly assigned four of the hand-written statements and their partnering interview transcripts and followed the same coding procedure. Across the two raters there was only one minor discrepancy (i.e., in one statement one rater scored 28 for consistent person details whereas the other rater scored 29). Thus, the overall inter-rater reliability agreement across all the thirty-six independent variables within the four statements was 99.3%.

## Results and Discussion

All 15 final statements contained errors in that their content diverged from the original verbal account provided by the witness in at least one of the ways captured by our coding protocol. Descriptive results across each of the 15 statements are presented in Table 1. Consistent detail percentages ranged from 19.28 to 86.97%. Known facts accounted for 1.30–20.00% of the statements. The most commonly observed type of error were omission errors which ranged from 4.76% of a statement to 51.81%, followed by distortions ranging from 1.85 to 19.28% of a statement. The intrusion of new (i.e., previously unmentioned) information had a range of 0.00–20.51% of details. Only two statements did not include any intrusion errors. Finally, three statements contained contradictory information (range 0.00–5.00%). Examples of each error category observed in statements are presented in Table 2.

**TABLE 1:** Number of details per interview transcript and hand-written statement pair across coded consistency category (% of transcript); illustrates discrepancy across each of the fifteen statements.

Consistency category	1	2	3	4	5	6	7	8	9	10	11	12	13	14	15
Consistent details	102 (69.39)	74 (63.25)	95 (58.64)	99 (43.04)	130 (68.78)	96 (41.03)	121 (66.12)	249 (72.59)	37 (52.86)	320 (85.33)	267 (86.97)	245 (71.33)	16 (19.28)	167 (60.51)	347 (67.77)
Known details	8 (05.44)	9 (07.69)	7 (04.32)	3 (01.30)	7 (03.70)	26 (11.11)	27 (14.75)	33 (09.62)	14 (20.00)	6 (01.60)	10 (03.26)	30 (08.72)	6 (07.23)	10 (03.62)	20 (03.91)
Intrusions	16 (10.88)	7 (05.98)	1 (00.62)	9 (03.91)	22 (11.64)	32 (13.68)	3 (01.64)	8 (02.33)	0 (00.00)	3 (00.80)	0 (00.00)	25 (07.27)	2 (02.41)	19 (06.88)	105 (20.51)
Distortions	14 (09.52)	6 (05.13)	3 (01.85)	17 (07.39)	8 (04.23)	15 (06.41)	9 (04.92)	46 (13.41)	6 (08.57)	8 (02.13)	8 (02.61)	20 (05.81)	16 (19.28)	21 (07.61)	12 (02.34)
Contradictions	0 (00.00)	0 (00.00)	0 (00.00)	0 (00.00)	0 (00.00)	0 (00.00)	1 (00.55)	5 (01.46)	0 (00.00)	0 (00.00)	0 (00.00)	0 (00.00)	0 (00.00)	4 (01.45)	0 (00.00)
Omissions	7 (04.76)	21 (17.95)	56 (34.57)	102 (44.35)	22 (11.64)	65 (27.78)	22 (12.02)	2 (00.58)	13 (18.57)	38 (10.13)	22 (07.17)	24 (06.98)	43 (51.81)	55 (19.93)	28 (05.47)
Total details	147	117	162	230	189	234	183	343	70	375	307	344	83	276	512

**TABLE 2:** Examples of discrepancies across the interview transcripts and hand-written statements.

Consistency category	Interview transcript—verbal evidence	Hand-written statement—written evidence
Distortions	1. “Few of the lads.” 2. “One of them” (carrying TV).	1. “Gang of youths.” 2. “They were carrying TV.”
Contradictions	1. “Couldn’t hear what was being said.”	1. “I recall the conversation during this.”
Omissions	1. “Car was definitely a Metro.” 2. “I didn’t actually see any damage.” 3. “No caps, no glasses on youths.”	1, 2, and 3 omitted from written evidence.
Intrusions	1 and 2 not mentioned by the witness during the interview.	1. “There were no obstructions to my view.” 2. “Brown hair.”

To summarize, every statement examined contained errors, primarily omissions, followed by distortions and then intrusions (new) information. Thus, in this sample, the evidential product (i.e., the witness statement) was never an exact replication of what the witness actually said at interview. Worse, in some cases there were sizeable discrepancies between the original verbal account provided by the witness and what the officer recorded in the statement. There are a number of possible reasons for such discrepancies. First, there are significant cognitive demands associated with both interviewing and statement-taking. Recent research by Hanway et al. (2021) observed that when people complete tasks intrinsic to investigative interviewing (such as listening, remembering, judging the information provided, and generating follow-up questions to ask) not only do they experience a higher cognitive burden than those who simply have to listen to a witness’s statement, but they also make more recall errors when asked to recall what the witness actually said. Further, research examining memory for conversation has found that it tends to be gist as opposed to verbatim due to competing demands (Brown-Schmidt and Benjamin, 2018). In the current sample of statements, recall errors may well be reflected in the omission errors (information the interviewer did not remember when writing the statement) and distortion errors (information the interviewer remembered incorrectly when writing the statement). Second, when writing the statement, information that fits with an existing schema for the reported event (e.g., an archetype; Shepherd and Milne, 1999) may have inadvertently been prioritized over non-schematic information, particularly when cognitive resources were limited. Finally, some discrepancies may reflect the preconceptions or beliefs officers hold about what constitutes “a good statement” and what information is relevant or appropriate to include. In such instances, officers may have edited or distorted the information accordingly.

Thirteen of the statements included information that was not mentioned by the interviewee. In other words, “new” intruded information (beyond known facts) was introduced by the officer when writing the statement. This new information may be the result of a source monitoring error whereby the officer misremembered the original source of the information and accidentally attributed it to the witness interview when in fact the information was obtained elsewhere (e.g., another witness; see Source Monitoring Framework; Johnson et al., 1993; Hanway, 2021). As the number of witnesses the interviewer deals with increases, this type of error is likely to be more prevalent. It could also be the case that interviewers incorporate this “new” information to increase the plausibility of the witness’s account. Indeed, visually recorded police interviews (often used as evidence in chief) are regularly critiqued by legal practitioners for not being succinct and not taking the form of a coherent chronological narration (Westera et al., 2017). Worryingly, interviewers striving for a “good” statement in the eyes of the justice system may result in evidence that is distorted, has intrusions, and with omissions. Future research should further explore the extent to which preconceptions about what constitutes a “good statement” and how any pre-existing beliefs distort the production and evaluation of statements and other evidence. For these reasons, psychological, legal, and linguistic professionals alike have criticized the justice system for an over-reliance on the statement-taking process as it lacks accuracy, legitimacy, and transparency (e.g., Heaton-Armstrong and Wolchover, 1992; Milne and Shaw, 1999; Rock, 2001; Westera et al., 2011).

To examine the nature of the errors in more depth, errors were examined with respect to detail type (person, action, object, surrounding, conversation, and temporal). Means (and SDs) across detail type were calculated for each variable (omissions, distortions, and intrusions) across the 15 statements. Only three statements contained contradictory information. An example can be seen in Table 2 and worryingly it concerned witness reliability with regard their visibility at the time the event was witnessed. Table 3 shows that every type of detail was omitted and this occurred in each of the 15 pairings. Omission errors primarily pertained to the objects and people involved in the incident and their actions. Distortions also primarily concerned the people in the events, where the event took place, what people did, and the objects involved. With respect to intrusions, the largest mean number of these errors related to the objects involved, followed by the key players in the incidents and what they did. In sum, errors identified in this sample pertained to forensically relevant details, including information about the perpetrator, their actions, where the incident took place and the objects used.

**TABLE 3:** Means and standard deviations for consistency categories by detail type across interview-statements (*N* = 15).

	Consistent details *M* (*SD*)	Known details *M* (*SD*)	Intrusions *M* (*SD*)	Distortions *M* (*SD*)	Contradictions *M* (*SD*)	Omissions *M* (*SD*)
Person	41.80 (30.93)	4.13 (6.58)	4.13 (6.58)	4.13 (3.48)	0.27 (0.59)	9.33 (7.86)
Action	35.00 (23.24)	2.00 (2.98)	3.53 (5.89)	3.27 (3.63)	0.20 (0.56)	7.93 (7.78)
Object	40.87 (24.85)	1.93 (2.40)	5.47 (13.25)	2.40 (1.92)	0.07 (0.26)	8.67 (4.78)
Surroundings	28.53 (27.19)	3.33 (2.47)	2.20 (2.88)	2.93 (2.79)	0.07 (0.26)	5.80 (7.70)
Conversations	2.47 (4.22)	0.07 (0.26)	0.07 (0.26)	0.20 (0.77)	0.00 (0.00)	0.93 (1.75)
Temporal	9.00 (9.45)	3.60 (2.64)	1.40 (1.76)	1.00 (1.36)	0.07 (0.26)	2.00 (2.67)

Overall, given that this exploratory work identified clear discrepancies in all of the statements that were examined with reference to the original account given by the witness, it appears this issue may be relatively commonplace. If that is indeed the case, what are the implications? First, given that the criminal justice system relies on accurate witness statements to both pursue investigations and inform subsequent legal decision making, statements that contain errors of any kind may not only result in wasted time and resources but also jeopardize the pursuit of justice. Secondly, given that cases can take some time to come to court, witnesses may rely on reviewing their statement before testifying. If that statement contains erroneous information, then it is entirely possible that the witness’s memory of their original experience will be distorted accordingly (e.g., Misinformation Effect; see Frenda et al., 2011, for a review).

There is a simple solution to address such concerns: visually record all evidence gathering interactions, harnessing technology, such as a body-worn video recording device, to legitimize the process and allow reliability assessment. Indeed, some jurisdictions now favor visually recording the process, especially for vulnerable groups (Davies et al., 2016). However, a move toward more accurate witness testimony through visual recording also requires an understanding and adoption of basic memory principles (i.e., that memory is both fallible and easily contaminated) and that the written statement is not the verbatim record it was previously assumed to be. In addition, the raw product of memory, such as recall, may not emerge in the form of a chronologically narrated, comprehensively detailed story. Nonetheless, allowing the witness to provide their own account in their own words, is more likely to provide accurate investigative and evidential information compared to a non-transparent, ill-monitored, translational process such as statement-taking.

This preliminary project examined a small sample of cases and, although consistent with the case samples examined by previous researchers (e.g., McLean, 1995; Lamb et al., 2000; Hyman Gregory et al., 2011), further work is necessary to examine this issue across a larger case sample involving different case types. For instance, it may be the case that certain case types are more prone to some of the translational issues we observed in the current sample. Indeed, there are potentially a multitude of factors that could influence the statement taking process (such as training regimes, method trained for interviewing witnesses and so on). Notably, however, every statement in our sample contained errors—a finding that is also consistent with previous research (McLean, 1995). It is also important to note that the interview-statement pairings were from the 2001 evaluation study, however, there has been almost no research or practice change since that time with regard the production of witness statements, and thus the results are reflective of current practice. Many countries also do not electronically record their interviews/interrogations with suspects, instead a written report is produced (e.g., the Netherlands). A limited amount of work has started to look at the accuracy of this written report and has similarly found omission errors (e.g., Malsch et al., 2018). For example, Malsch et al. (2018) found that only 24% of all spoken words were accounted for in the reports, though this included interviewer and interviewee utterances. More research is urgently needed in this area.

To conclude, in the current study, a comprehensive coding protocol allowed us to determine that the errors identified in this sample in the form of omissions, distortions, and intrusions, pertained to forensically relevant details. In all fifteen statements there were errors, across all detail types, though there was a lot of variability across the statements. Omission errors were the most frequently observed error. Thus, due to cognitive demands of the multi-faceted interviewing task, errors will emerge. Perhaps it is time to acknowledge that, despite their importance within the criminal justice system, statements generated in this translational way are likely error-ridden as a result of imperfect human cognition and that technological solutions should take precedence.

## Data Availability Statement

Due to the private/sensitive nature of the material, the datasets presented in this article are not readily available. Requests to access the datasets should be directed to the lead author.

## Ethics Statement

The studies involving human participants were reviewed and approved by the Home Office funded research permission and Metropolitan Police permission, and complied with the University of Portsmouth Ethics procedure at that time. Written informed consent for participation was not required for this study in accordance with the national legislation and the institutional requirements.

## Author Contributions

RM was PI on the project, developed coding system, attained the data, helped code and score, analyzed the data, and wrote the manuscript. JN coded and scored data set, analyzed the data, and helped write the manuscript. LH helped design the coding system and edited manuscript. JH coded and scored data. CC attained data set in the first place. All authors contributed to the article and approved the submitted version.

## Conflict of Interest

The authors declare that the research was conducted in the absence of any commercial or financial relationships that could be construed as a potential conflict of interest.

## Publisher’s Note

All claims expressed in this article are solely those of the authors and do not necessarily represent those of their affiliated organizations, or those of the publisher, the editors and the reviewers. Any product that may be evaluated in this article, or claim that may be made by its manufacturer, is not guaranteed or endorsed by the publisher.
